# Light-Driven [FeFe] Hydrogenase Based H_2_ Production in *E. coli*: A Model Reaction for Exploring *E. coli* Based Semiartificial Photosynthetic Systems

**DOI:** 10.1021/acssuschemeng.2c03657

**Published:** 2022-08-11

**Authors:** Marco Lorenzi, Mira T. Gamache, Holly J. Redman, Henrik Land, Moritz Senger, Gustav Berggren

**Affiliations:** † Department of Chemistry - Ångström, Molecular Biomimetics, Uppsala University, Lägerhyddsvägen 1, 75120 Uppsala, Sweden; ‡ Department of Chemistry - Ångström, Physical Chemistry, Uppsala University, Lägerhyddsvägen 1, 75120 Uppsala, Sweden

**Keywords:** Hydrogen, Hydrogenase, Whole-cell catalysis, Enzyme catalysis, Whole-cell spectroscopy, Semiartificial photosynthesis, ANOVA, Multivariate analysis

## Abstract

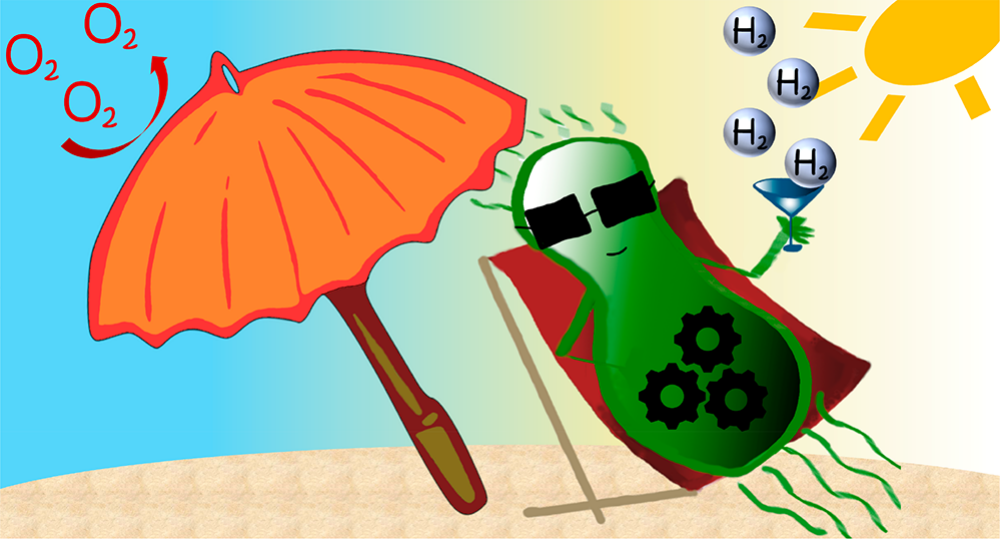

Biohybrid technologies like semiartificial photosynthesis are attracting increased attention, as they enable the combination of highly efficient synthetic light-harvesters with the self-healing and outstanding performance of biocatalysis. However, such systems are intrinsically complex, with multiple interacting components. Herein, we explore a whole-cell photocatalytic system for hydrogen (H_2_) gas production as a model system for semiartificial photosynthesis. The employed whole-cell photocatalytic system is based on *Escherichia coli* cells heterologously expressing a highly efficient, but oxygen-sensitive, [FeFe] hydrogenase. The system is driven by the organic photosensitizer eosin Y under broad-spectrum white light illumination. The direct involvement of the [FeFe] hydrogenase in the catalytic reaction is verified spectroscopically. We also observe that *E. coli* provides protection against O_2_ damage, underscoring the suitability of this host organism for oxygen-sensitive enzymes in the development of (photo) catalytic biohybrid systems. Moreover, the study shows how factorial experimental design combined with analysis of variance (ANOVA) can be employed to identify relevant variables, as well as their interconnectivity, on both overall catalytic performance and O_2_ tolerance.

## Abstract 2

Semiartificial photosynthetic H_2_ production in *E. coli* investigated via multivariate analysis to determine parameters controlling catalytic performance and O_2_ tolerance.

## Introduction

In order to tackle our current environmental issues and energy shortage, molecular hydrogen (H_2_) is considered a promising future energy vector, capable of driving the coming energy transition. Several approaches have been explored to realize such a hydrogen-powered society. Nature’s H_2_ producers, the hydrogenases, are intensively studied as alternatives to precious metal catalysts due to their capacity to utilize base metals (Ni and Fe) to enable high turnover frequency catalysis at low overpotential.^1^ [FeFe] hydrogenases in particular stand out as the most active, with reported TOFs exceeding 10^4^ s^–1^.^2^ These enzymes owe their remarkable activities to their unique active site, the H-cluster. The H-cluster consists of an organometallic diiron complex ([2Fe]_H_) linked to a canonical [4Fe4S] cluster through a bridging cysteinate.^2−5^

[FeFe] hydrogenases can be found in many different microorganisms, including photosynthetic green algae. Photobiological H_2_ production, albeit promising from a sustainability perspective, is limited by the low overall efficiency of natural photosynthesis.^6−9^ This limitation has triggered the parallel development of biohybrid devices where enzymes are coupled with synthetic photosensitizers, achieving higher solar energy-to-product efficiencies.^10−15^ However, the need to express and purify the enzymes in large quantities have made the scale-up of these systems challenging. Additionally, the high O_2_ sensitivity of [FeFe] hydrogenases represents a major limitation.^16−19^ Consequently, direct practical applications for this class of enzymes have remained limited in a solar fuel context.^14^ Employing whole-cell biocatalysts provides a path to overcome the two latter limitations, as it removes the need for expensive purification, and the metabolic activity of aerobic microorganisms can potentially shelter oxygen-sensitive enzymes. When combined with artificial light-harvesters, to yield semiartificial photosynthesis, the limitations of natural photosynthesis can also be alleviated.

Thus, semiartificial photosynthetic systems provide the possibility to combine the unrivaled catalytic power and self-healing capacity of biocatalysis with the efficiencies of artificial light harvesters.^20,21^ Over the past few years, a number of such systems have been reported. For example, intracellular gold nanoparticles have been used to drive the Wood–Ljungdahl Pathway in *M. thermoacetica* to fix CO_2_ into acetate,^22^ and Cd/S nanoparticles precipitated over the membrane of *M. barkeri* cells allowed for direct CO_2_-to-CH_4_ conversion.^23^ The organic dye eosin Y (and structurally related substances) has been combined with *S. oneidensis* cells to produce different small molecules including H_2_.^24^ Similarly, eosin Y has also been combined with *E. coli* cells to drive various enzyme catalyzed reactions,^25,26^ including hydrogenase catalyzed H_2_ production.^27^ Despite increasing interest in semiartificial photocatalytic systems, their optimization remains challenging due to the complex interplay between not only light harvester and enzyme catalyst, but also of parameters influenced by cell metabolism and homeostasis. Similarly, mechanistic insight generally remains limited due to the multicomponent nature of the systems.

**Figure 1:**
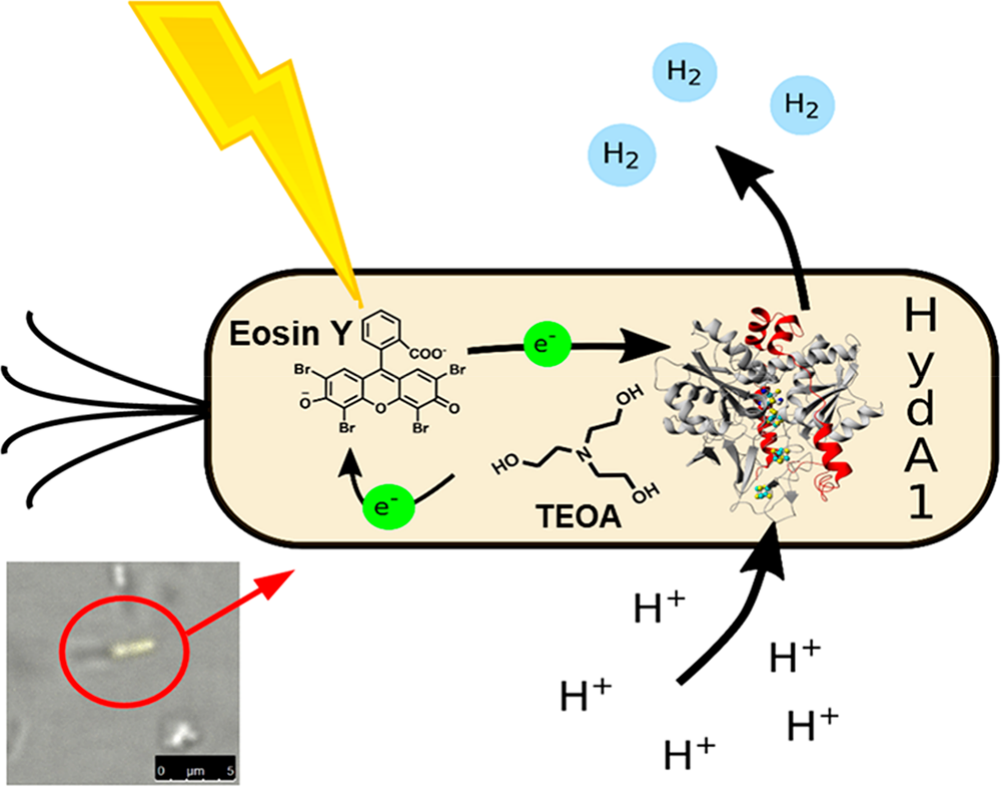
Graphical representation of the whole-cell photocatalytic system. Upon photoexcitation, eosin Y facilitates the electron transfer between TEOA and HydA1, which ultimately produces H_2_ gas. (inset) Fluorescence microscopy picture of an eosin Y stained *E. coli* culture. The picture shows a single focus plane. Additional fluorescence microscopy pictures are available in the Supporting Information (Figure S1).

In order to construct a model system to explore general aspects of such whole-cell biohybrid assemblies, we have taken advantage of our capacity to generate functional [FeFe] hydrogenases inside a cellular envelope via whole-cell artificial maturation.^5,28−30^ The relatively high concentrations of active enzyme obtainable via artificial maturation enable both spectroscopic and functional characterizations. Moreover, producing the active enzyme at a defined time-point allows us to evaluate its intrinsic stability, without the additional possibility of catalyst regeneration. We utilize this procedure to construct a light-driven *E. coli* based whole-cell biohybrid system, in which eosin Y is used to drive H_2_ production from heterologously expressed [FeFe] hydrogenase (Figure 1). A similar system was recently reported by Honda and co-workers and shown to significantly outperform analogous systems based on inorganic light harvesters.^27^ We employ spectroscopy to verify that the photosensitizer is able to transfer electrons to the [FeFe] hydrogenase inside the cells. Subsequently, the photocatalytic system is studied using a factorial design approach to identify variables and estimate their relevance, as well as to determine the interactions among variables.^31^ Finally, we use our highly oxygen-sensitive model catalyst to explore the protection granted by the cellular environment toward oxygen exposure.

## Results and Discussion

### Construction of the Whole-Cell Photocatalytic System

The choice of catalyst fell on an *E. coli* Bl21 strain expressing the model algal [FeFe] hydrogenase from *Chlamydomonas reinhardtii* (*Cr*HydA1). As *E. coli* lacks the enzyme machinery required for synthesis and insertion of the [2Fe]_H_ complex,^32,33^ the strain expresses *Cr*HydA1 in its apo form. Subsequently, the enzyme was artificially matured *in vivo* following an established protocol to yield the fully functional holo-enzyme in the cytoplasm at a given time-point.^29^ Eosin Y and triethanolamine (TEOA) were chosen as photosensitizer and sacrificial electron donor, respectively, as both of these compounds have been used before to drive whole-cell photocatalysis.^24,25,27^ Eosin Y was chosen over other commonly employed photosensitizers due to its reported capacity to drive photoreduction of [FeFe] hydrogenases and other related enzymes *in vitro*.^35−37^ Moreover, as eosin Y is employed as a cytoplasmic staining agent,^38^ a high level of membrane penetration was expected, which could circumvent the need for an additional cell permeable redox mediator.^39,40^ Indeed, confocal fluorescence microscopy verified that eosin Y fluorescence was localized inside of the *E. coli* cytoplasm (Figures 1 (inset) and S1). Upon illumination, the whole-cell photocatalytic system containing the active holo-enzyme as well as eosin Y (100 μM) and TEOA (100 mM) was found to be able to produce up to ∼0.5 μmol ml^–1^ OD_600_^–1^ of H_2_ over the course of 24 h, in line with earlier reports.^27^ Cell integrity was monitored over the course of the photocatalytic reaction by verifying the absence of active hydrogenase in the supernatant, via *in vitro* H_2_ production assays as previously described.^28,30^ Even after 24 h, the supernatant displayed only trace activities as compared to the whole-cell fraction (≤5% relative activity, Figure S2). However, plating experiments showed that exposing the cells to the photocatalytic conditions significantly impaired their viability (Figure S2). The light-driven system greatly outperformed the fermentative H_2_ productivity observed in the presence of glucose for *E. coli* cells containing artificially maturated *Cr*HydA1.^28,41^ Conversely, incubation of parallel samples in darkness, or illuminating samples lacking any of the key components, *i.e.*, the enzyme, eosin Y, or TEOA, resulted in significantly lower H_2_ accumulation (Figure S3).

**Figure 2:**
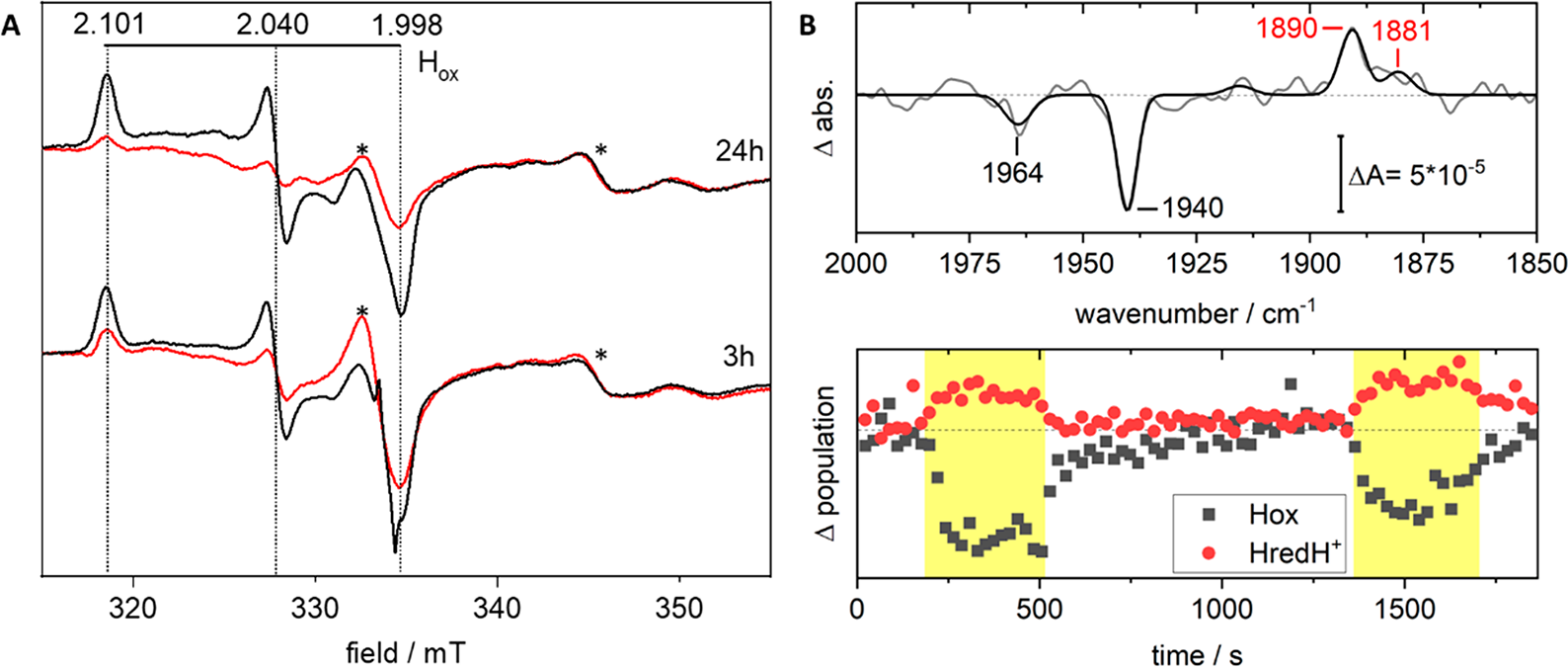
Assembly of the H-cluster and its photoreduction verified by EPR and ATR-FTIR spectroscopy. (A) EPR spectra recorded on *Cr*HydA1-containing cell suspensions following light or dark incubation in the presence of eosin Y (100 μM) and TEOA (100 mM). Samples were collected after either 3 or 24 h of illumination (red traces) or dark incubation (black traces). In all samples, the only distinct H-cluster derived EPR signal is attributable to an H_ox_ state (*g*_*xyz*_ = 2.101 2.040 1.998, indicated with horizontal bar), with no signs of degradation or inhibition after 24 h. Upon illumination, samples show a less intense signal, compatible with the formation of the EPR-silent state H_red_H^+^. Prominent contributions from the whole-cell background are indicated with asterisks. EPR experimental conditions: *T* = 10 K, *P* = 1 mW, ν = 9.28 GHz. (B, top) Difference ATR-FTIR spectra of a rehydrated film of *E. coli* cells containing *Cr*HydA1, eosin Y, and TEOA recorded before and after *in situ* illumination. The difference spectrum (data gray, fit black) shows the disappearance of the oxidized state (H_ox_, marker bands at 1964 and 1940 cm^–1^) and the simultaneous appearance of bands attributable to reduced H-cluster states of *Cr*HydA1 (H_red_H^+^ and H_sred_H^+^, marker bands at 1890 and 1881 cm^–1^, respectively), verifying photoreduction inside the *E. coli* cells. Spectra prior to baseline correction are shown in Figure S4. (B, bottom) Redox state population monitored over time, via the area of the marker bands. During the illumination periods (yellow boxes), reduced states accumulate.

In order to verify the involvement of the heterologously expressed hydrogenase in the photocatalytic reaction, the system was characterized through a combination of electron paramagnetic resonance (EPR) and attenuated total reflection Fourier transformed infrared (ATR-FTIR) spectroscopy. X-band EPR spectra were recorded on whole-cell suspensions, collected after 3 and 24 h of incubation in complete darkness or exposed to continuous illumination, in the presence of eosin Y and TEOA (Figure 2A). In EPR spectra recorded for all four conditions, the only discernible H-cluster signal was attributable to the oxidized active-ready resting state H_ox_ (*g*_*zyx*_ = 2.101 2.040 1.998).^1,2,42^ Illumination of the cell suspensions resulted in a significant decrease in amplitude of the rhombic H_ox_-signal, relative to the corresponding samples incubated in darkness. This observation is in line with the formation of reduced, EPR-silent, H-cluster states, *e.g.*, H_red_ or H_red_H^+^.^42^ Additionally, samples illuminated for 24 h displayed only a minor decrease in signal intensity, as compared to samples illuminated for 3 h. The absence of the CO inhibited state, H_ox_-CO, is also noteworthy. The latter state is expected to form if a significant fraction of the H-cluster population degrades, and it is commonly formed upon irradiation of [FeFe] hydrogenases by white light.^43,44^ Thus, under the given conditions, the H-cluster is not significantly damaged by continuous illumination. ATR-FTIR spectroscopy was employed to probe the CO and CN^–^ region of the spectrum, where the spectroscopic features of several H-cluster states are well established.^1,2,42^ The FTIR data further supported the presence of the H_ox_ state under dark incubation, with detection of its most intense reporter bands (at 1940 and 1964 cm^–1^). Due to the low relative concentration of *Cr*HydA1 in the *E. coli* cells, a complete spectroscopic fingerprint was not obtainable. Critically, the reduction of H_ox_ to the one-electron reduced state H_red_H^+^ (reporter band at 1890 cm^–1^) was readily observable upon illumination on a time-scale of seconds (Figure 2B).^42^ A small population of the two-electron reduced state H_sred_H^+^ was also discernible in the difference spectra, from a positive band at 1881 cm^–1^ (Figure 2B). Evidently, eosin Y is capable of driving the photoreduction of the [FeFe] hydrogenases present in the *E. coli* cells, analogously to what has been observed with the purified enzyme before.^35,36^ In combination, these observations strongly support the notion that the observed H_2_ production is attributable to the semisynthetic [FeFe] hydrogenase and that the system displays a high level of stability even on a day time-scale (see also Figure S2).

### Identification of Key Variables and Their Interconnectivity

A whole-cell photocatalytic system is composed of several strongly interconnected elements, which are expected to result in nonlinear variable dependence. Thus, a multivariate approach was employed in the experimental design and analysis. This allowed the possibility of working with a relatively small data set, while being able to account also for the combined effect of two or more variables. Four main variables were selected: cell concentration (**OD**_**600**_), eosin Y concentration (**EY**), pH value (**pH**), and light intensity (**LightT**). While **OD**_**600**_, **EY**, and **pH** were assigned two levels (defined as −1 and 1), **LightT** was given three (defined as −1, 0, and 1). The three-level variable (**LightT**) can be inserted in a two-level design by treating it as a combination of two two-level variables (**Light1** and **Light2**). The resulting variables scheme is presented in Table 1 and yielded 32 total runs (2^5^). This included 24 unique runs and eight technical replicates that allow for a better estimation of internal error and statistical significance (see Table S1 for a detailed summary of the respective samples). The same combinatorial scheme was applied to two separate sets of samples, one prepared in a strict oxygen-free atmosphere and an equivalent set in which the samples were exposed to a 5% oxygen atmosphere.

**Table 1:** Variables for the Design of the Experiment and Their Assigned Levels

OD_600_^a^	EY^b^	pH^c^	LightT^d^
1 (−1)	10 μM (−1)	6.5 (−1)	2500 lx (−1)
			4000 lx (0)
5 (+1)	100 μM (+1)	7.5 (+1)	5000 lx (+1)

a Cell concentration, as determined from absorbance at 600 nm.

b Eosin Y concentration.

c Initial pH (phosphate buffer, 100 mM).

d Light intensity, in lux. The value given to each variable in the analysis of variance (ANOVA) is shown in parentheses.

**Figure 3:**
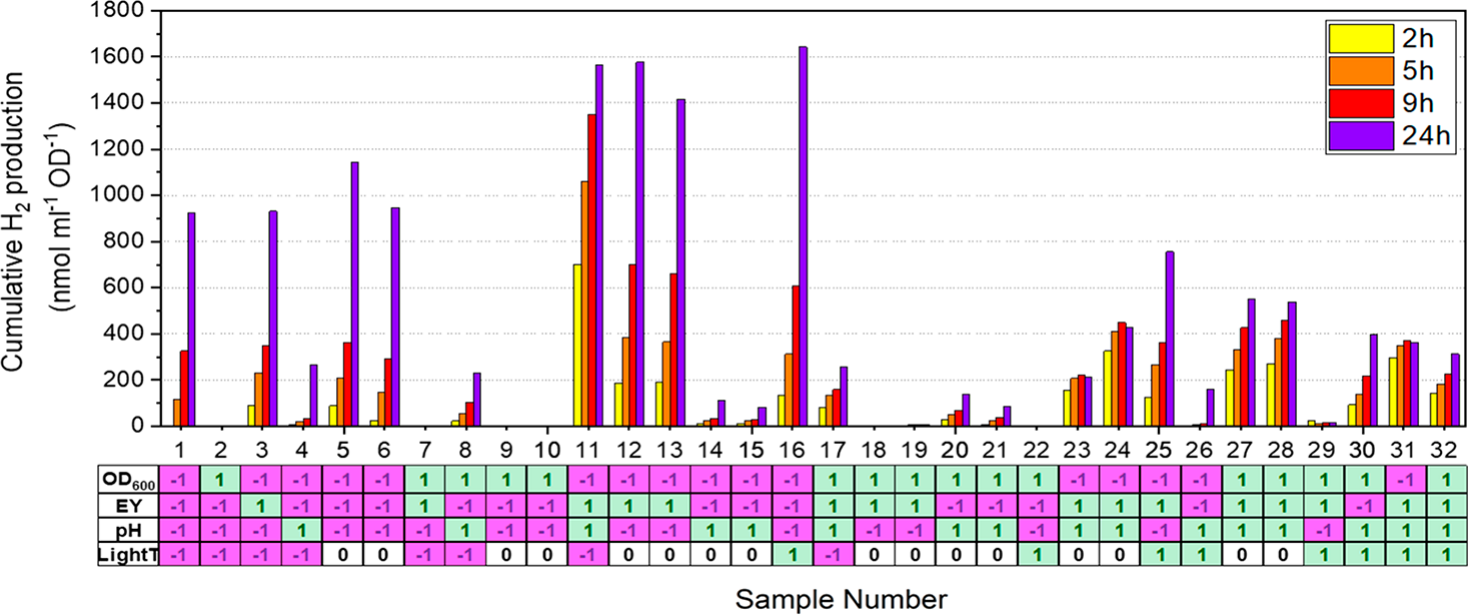
Photocatalytic H_2_ production from samples representing the different combinations of variables in the oxygen-free set. Cumulative H_2_ production is expressed as nmol ml^–1^ OD_600_^–1^. For each sample, data is shown for H_2_ produced after 2 h (yellow bars), 5 h (orange bars), 9 h (red bars), and 24 h (blue bars) of illumination. The Sample Number (1–32) refers to a specific combination of variables as defined in the bottom table: +1 (green); 0 (white); −1 (pink). See Tables 1 and S1 for additional details.

For the oxygen-free set, samples corresponding to the different variable combinations were anaerobically prepared in sealed glass vials and exposed to light. The cumulative H_2_ production at selected time points (2, 5, 9, and 24 h) was then determined and reported as specific H_2_ production (i.e., nmol H_2_ ml^–1^ OD_600_^–1^). As seen in Figure 3, large variations in H_2_ production are observed for the different samples, with final specific H_2_ production yields varying from 0 to 1600 nmol ml^–1^ OD_600_^–1^. It is immediately apparent that specific variable combinations can be identified as favorable for high specific H_2_-productivity. The four peak producers, samples 11–13 and 16, all share a high eosin Y concentration (**EY**) combined with a low cell density (**OD**_**600**_). If samples are instead evaluated based on apparent quantum yield, the high cell-density sample 27 and its technical replicate 28 stand out, displaying a full-spectrum apparent quantum yield of 1.1% over the first 5 h of production (at 4000 lx). Although their specific H_2_ production is lower than several low cell concentration samples (e.g., samples 11–13), the total amount of hydrogen produced by these samples is higher and reflects a more efficient use of light. Moreover, different samples evidently show different production profiles over time, with some displaying a marked slowdown in production after the first 5–9 h (e.g., samples 11, 23, and 24). In all, this confirms that the chosen variables have an effect on the H_2_ production capabilities of the photocatalytic system both in terms of “initial rate” and long-term stability.

To identify the real variables of interest and their reciprocal interaction effects, the data set was then subjected to an analysis of variance (ANOVA). An ANOVA analysis is an inferential statistics method that allows for comparing the mean values of groups of samples and evaluate whether these groups significantly differ between each other. Samples in a data set can be grouped based on the assigned values of one or more variable(s). As the number of groups taken into consideration increases, *e.g.*, by grouping based on high-order combination of variables, ANOVA becomes a very convenient method for managing this type of data analysis. A model including second- and third-order interactions was applied to the 5 and 24 h time points, to also elucidate potential variation over time (Tables 1, S2, and S3). The effect of single variables and their combinations can be investigated both in terms of magnitude of the observed effect—measured as amount of variation attributable to each source—and in terms of statistical significance. Statistical significance is expressed with a *p-value* coming from a null hypothesis significance test, representing the compatibility between the observation and the null hypothesis. A threshold value (α) of <0.05 is conventionally used and was adopted herein.

The analysis of the 5 h H_2_ production shows an internal error limited to ∼8% of the total variance in the data set and statistical relevance for the effect of **OD**_**600**_ (*p* = 0.0009), **EY** (*p* = 0.0007), and **pH** (*p* = 0.0405) (Tables 2 and S2). The light intensity (**LightT**) instead seems to be almost noninfluential. Its effect alone explains only ∼1% of the total variance, and it is statistically not significant. This latter result indicates that there is a factor other than photon flux limiting H_2_ production. For higher-order interaction, the ANOVA shows relevance of the interaction of OD_600_ and eosin Y (**OD**_600_*EY, *p* = 0.0141); eosin Y and pH (EY*pH, *p* = 0.0042); and of OD_600_, pH, and light intensity (**OD**_600_*pH*LightT, *p* = 0.0343). This last observation suggests that the role of light intensity is in fact not negligible but strongly depends on the levels of other variables and therefore cannot be investigated in isolation.

**Table 2:** Influence of Main Variables and Selected Combinations in the Different Data Sets Studied by ANOVA^a^

	variable	OD_600_	EY	pH	LightT	OD_600_*EY	OD_600_*pH	EY*pH
5 h	*p*-value	0.0009	0.0007	0.0405	0.6641	0.0141	0.2558	0.0042
(anaerobic)	variance	270701	287490	68600	10581	109327	18023	168060
24 h	*p*-value	0	0.1224	0.0538	0.7617	0.3865	0.0002	0.0315
(anaerobic)	variance	2955729	143270	240199	28143	41260	1714877	314142
5 h	*p*-value	0.0628	0.0132	0.3472	0.2461	0.0341	0.0014	0.0241
(5% O_2_)	variance	1345	2899	288	969	1879	6635	2221

a Data sets include anaerobic samples at the 5 and 24 h time points and 5% oxygen exposed samples at the 5 h time point. Influence given as variance, and their associated *p*-value. See Tables S2–S4 for additional details.

**Figure 4:**
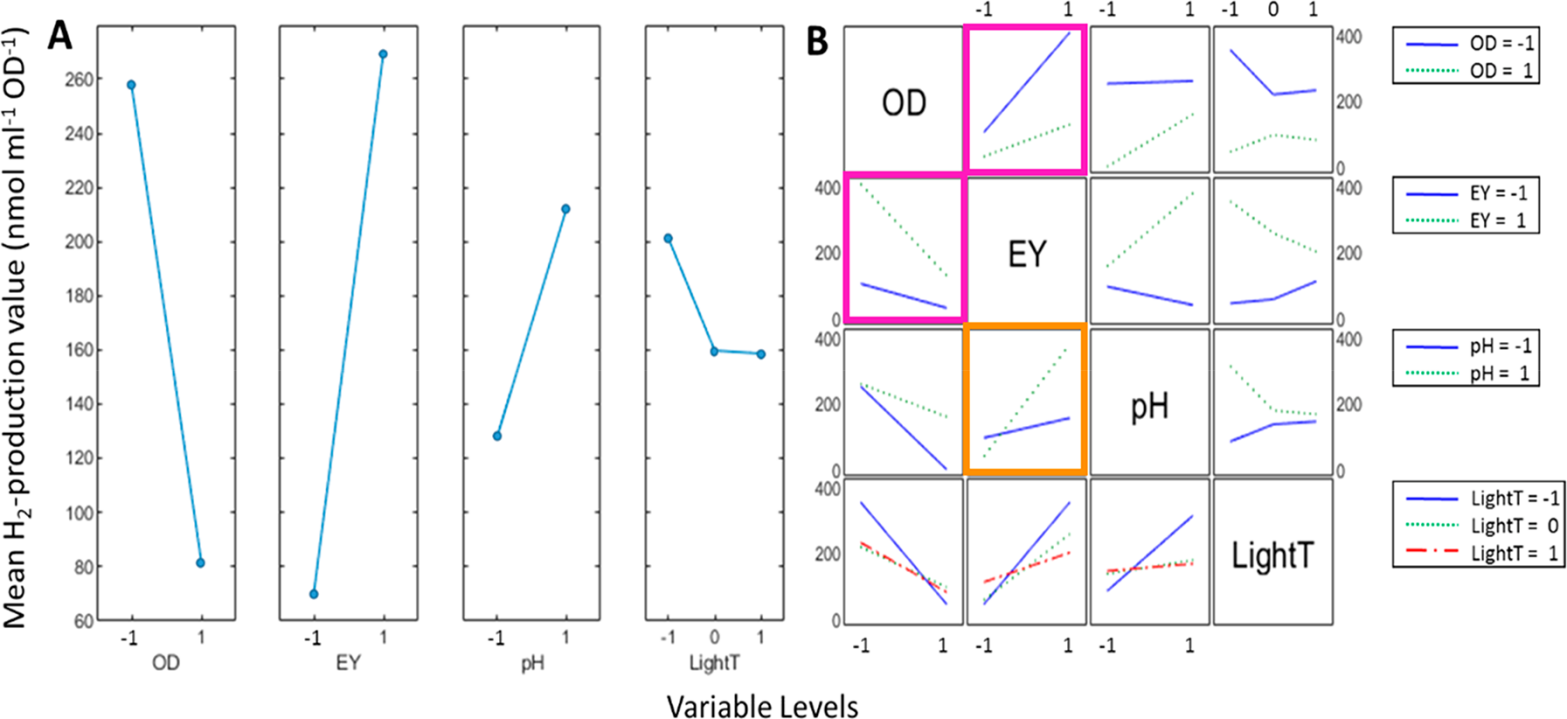
Main effects and interactions plot for the 5 h time point on the oxygen-free set. (A) The main effects plot visualizes the magnitude and the direction of the effect of varying the level of the single variables on the mean H_2_ production. (B) The interaction plot shows the effect of a single variable (columns, levels indicated on the *x*-axes) in relation to the level of another distinct variable (rows, levels represented with colored lines as indicated in the legends). See Table 1 for the definition of the variables. Selected boxes in panel B are color coded (for details, see the main text), and trend lines are added between data points as a visual guide.

The main effects plot (Figure 4A) is a visual representation of the correlation of each individual variable (**OD**_**600**_, **EY**, **pH**, and **LightT**) with the samples’ H_2_ production. The two most important main variables are **OD**_**600**_ and **EY**, as indicated by their relatively steep slopes. Increasing the amount of cells present in the reaction mix (**OD**_**600**_) has a strong detrimental effect in terms of specific H_2_ production. This effect is potentially due to a decreased light penetration in the sample caused by increased light scattering in relatively dense cell suspensions. Moreover, a higher concentration of eosin Y strongly correlates with higher productivity on a short time-scale. The variance and associated *p*-values shown in the ANOVA matrix (Tables 2 and S2) reveal that the interaction effect of the **OD**_**600**_ and **EY** variables also has to be considered (**OD**_600_*EY). The correlation between variables is shown in the interaction plot (Figure 4B). At the crossing of the two variables **OD**_**600**_ and **EY** (Figure 4B, purple boxes), we can see how a high cell density severely reduces the positive effect of an abundance of photosensitizer and how this negative interaction is smaller at low eosin Y concentrations. Evidently, increasing both catalyst amount (**OD**_**600**_) and eosin Y concentration (**EY**) yields diminishing returns with regard to promoting a high specific H_2_ production rate. This effect could be attributable to a reduced availability of eosin Y per cell in a dense cell suspension, suggesting that the amount of photosensitizer is the more important factor. The impact of pH on system performance is somewhat counterintuitive. Our data reveals that high pH correlates with higher H_2_ production, despite effectively corresponding to a lower substrate (proton) concentration (Figure 4A). *In vitro* assays have shown that *Cr*HydA1 has an optimal activity slightly below 7.^45,46^ However, a higher pH value increases the efficiency of TEOA as sacrificial electron donor.^47^ A related system employing the inorganic photosensitizer GaN:ZnO in place of eosin Y displayed an apparent pH optimum around 8.^48^ Moreover, the interaction plot shows that the pH effect is intertwined with the concentration of eosin Y (Figure 4B, orange box). In particular, we can see that at low pH values there is little advantage in increasing the amount of eosin Y in the reaction medium. This effect suggests differences in photochemistry, potentially including TEOA chemistry, or in photosensitizer uptake, requiring more detailed investigations to fully elucidate.

**Figure 5:**
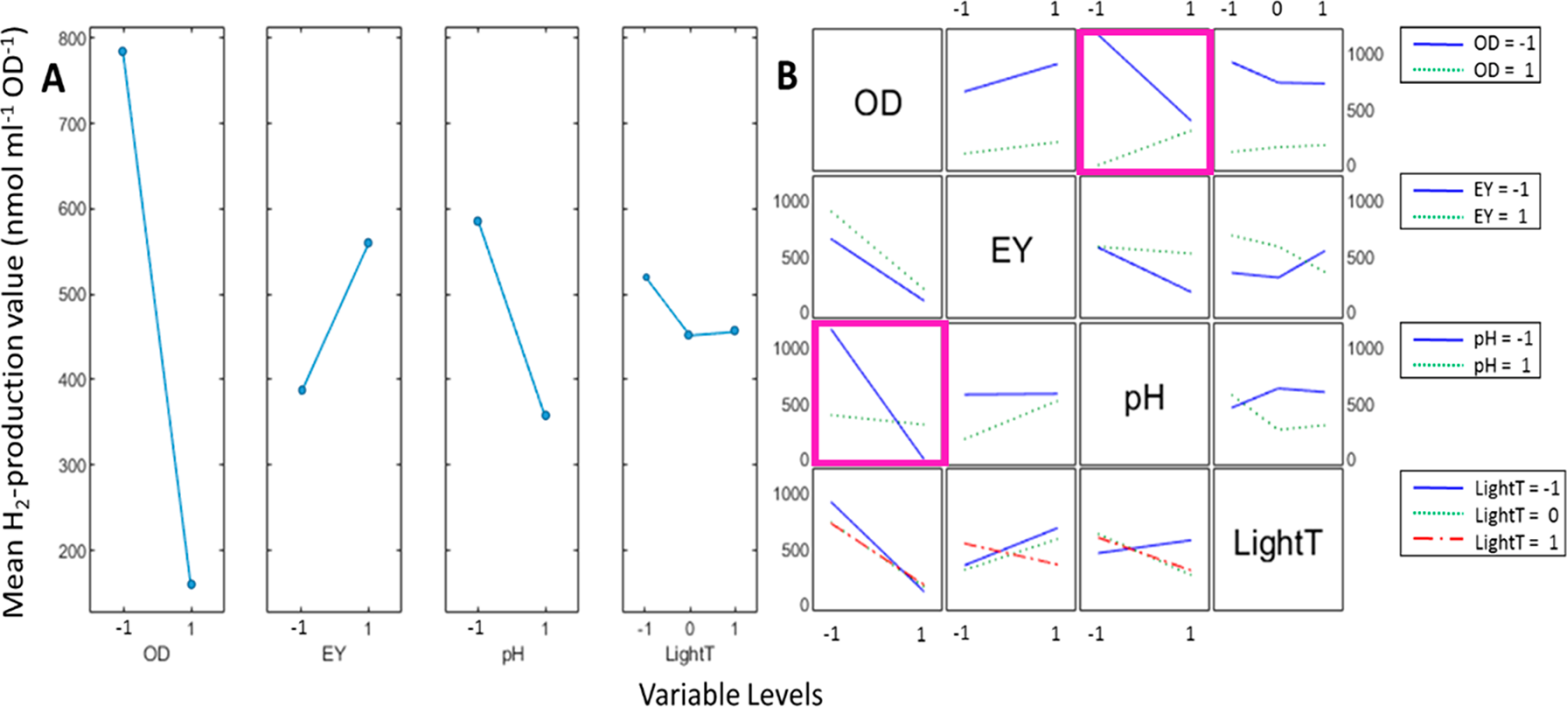
Main effects and interactions plot for the 24 h time point on the oxygen-free set. (A) The main effects plot visualizes the magnitude and the direction of the effect of varying the level of the single variables on the mean H_2_ production. (B) The interaction plot shows the effect of a single variable (columns, levels indicated on the *x*-axes) in relation to the level of another distinct variable (rows, levels represented with colored lines as indicated in the legends). See Table 1 for the definition of the variables. Selected boxes in panel B are color coded (for details, see the main text), and trend lines are added between data points as a visual guide.

When studying the effect of variables on the long-term (24 h) productivity of the system, it is found that cell density (**OD**_**600**_) and the interaction **OD**_600_*pH contributes almost 50% of the total variance (with *p*-values of <0.0001 and 0.0002, respectively) (Figure 5 and Tables 2and S3). Similar to the 5 h data, a higher cell density caused a significant drop in specific H_2_ production. The interaction plot provides a more detailed picture, as the negative effect of high cell density is striking at low pH values but becomes negligible at high pH (Figure 5B, purple boxes). The overwhelming importance of these two factors (**OD**_**600**_ and **OD**_600_*pH) highlights that the whole-cell system cannot be considered innocent, reflecting the interplay between the cells’ metabolism and reaction environment. Moreover, it is noteworthy that eosin Y concentration no longer appears to be a significant factor for H_2_ production on longer time scales.

### Oxygen Tolerance

To explore the effect of oxygen, samples were prepared analogously to the oxygen-free set. Subsequently, 25% of the vials’ headspace gas was replaced with air, yielding an atmosphere with ∼5% O_2_. No H_2_ production could be detected upon illumination directly following the addition of O_2_, as expected from complete irreversible inhibition of the enzyme, rapid O_2_ induced quenching of the photosensitizer, or a combination thereof.^16−19^ However, incubation of the cell suspensions in darkness following the O_2_ injection resulted in a significant drop in O_2_ concentration. This is attributable to the cellular respiration of the *E. coli* cells, and after 2 h, residual O_2_ was close to or below the detection threshold of the gas chromatograph (<0.15%). Illumination of these oxygen-exposed, and subsequently dark-incubated, cell suspensions revealed that H_2_ production could indeed be restored. Although H_2_ production was in most cases severely diminished, some samples retained moderate-to-low activities (Figure S5).

In particular, samples 1, 5, 6, and 16 display strikingly high activities. Interestingly, all of these samples are assigned the same values for all variables with the exception of light intensity (Table S1). This included a low cell density and eosin Y concentration, in combination with a low pH value, which suggests that these conditions might be particularly apt to protect the whole-cell catalyst from oxygen inactivation. The ANOVA (Tables 2 and S4 and Figure S6) revealed a strong interaction effect of culture density and pH (**OD**_600_*pH, *p* = 0.0014). Again, this highlights the importance of the cells’ metabolic state, which in turn is linked to their capabilities of consuming oxygen and dealing with reactive oxygen species (ROS). The only main variable with significant influence was **EY** (*p* = 0.0132). An increase in concentration of eosin Y strongly correlates with diminished H_2_ production capabilities, indicating that the photosensitizer has a role in enhancing oxidative damage. As our system is incapable of regenerating the active holo-enzyme, the activity observed postoxygen exposure directly verifies the intrinsic stability of the [FeFe] hydrogenase under these whole-cell conditions. This is in line with earlier reports that *E. coli* cells can protect [FeFe] hydrogenases from oxygen damage,^30^ a property attributable to the shielding and the oxygen scavenging provided by the cellular envelope.

## Conclusions

Herein, we report on an in-depth analysis of a semiartificial photosynthetic assembly, consisting of *E. coli* cells heterologously expressing an H_2_ producing enzyme, [FeFe] hydrogenase, combined with the organic photosensitizer eosin Y. The observed fluorescent staining and light-dependent H_2_ production confirms that eosin Y readily accumulates in the cytoplasm and that the system is functional. The involvement of the heterologously expressed [FeFe] hydrogenase in the photocatalytic process is verified spectroscopically. Remarkably, the system proved to be oxygen-tolerant despite the intrinsic oxygen sensitivity of *Cr*HydA1, highlighting the possibility of transforming *E. coli* into an oxygen-resilient photocatalytic system if a suitably O_2_-tolerant photosensitizer can be identified.

Furthermore, we demonstrate that a factorial design-of-experiment approach in combination with ANOVA is suitable to investigate complex photocatalytic systems, as readily controllable variables are enough to analyze the variance of H_2_ production. The relatively modest apparent quantum yields imply that there is ample space for optimizing electron transfer from the excited photosensitizer to the target enzyme. This could be achieved by using redox mediators or through optimization of the *E. coli* host strain, *e.g.*, using strains overexpressing the native redox partner for the enzyme, ferredoxins. The need to improve the bacterial host and to make the reaction medium more suited for a living cell is further underscored by the apparent loss of cell viability as well as the observation that, for long-term productivity, the most influential variables are closely related to the whole-cell catalyst (*i.e.*, cell density and pH).

In conclusion, the results and the methods applied to this H_2_ producing model system could be of interest for many other (photo)catalytic processes, which could benefit from an oxygen-resistant platform and a relatively simple experimental scheme for elucidating key parameters and leading optimization efforts.

## Supporting Information Available

Experimental details; a detailed sample scheme; ANOVA tables; and additional data related to fluorescence microscopy, H_2_ production, cell viability, and FTIR spectroscopy (PDF)
