# Supplementary material for: Light-Driven [FeFe] Hydrogenase Based H2 Production in E. coli: A Model Reaction for Exploring E. coli Based Semiartificial Photosynthetic Systems
Source: ACS Sustain Chem Eng. 2022 Aug 11;10(33):10760–7. doi: 10.1021/acssuschemeng.2c03657 (PMC9400101; doi:10.1021/acssuschemeng.2c03657)
Supplement: Supplementary file 1 — sc2c03657_si_001.pdf [file sc2c03657_si_001.pdf]

Supporting Information

Light-driven [FeFe] hydrogenase based H_2 production in *E. coli*: A model reaction for exploring *E. coli* based semi-artificial photosynthetic systems

Marco Lorenzi[1], Mira T. Gamache[1], Holly J. Redman[1], Henrik Land[1], Moritz Senger[2,*] and Gustav Berggren[1, *]

1) *Department of Chemistry - Ångström, Molecular Biomimetics, Uppsala University, Lägerhyddsvägen 1, 75120 Uppsala, Sweden; E-mail: Gustav.berggren@kemi.uu.se*
2) *Department of Chemistry - Ångström, Physical Chemistry, Uppsala University, Lägerhyddsvägen 1, 75120 Uppsala, Sweden; Email: Moritz.senger@kemi.uu.se*

Table of Contents

Experimental S2

Figure S1. Fluorescence microscopy pictures of eosin Y-stained *E. coli* cells. S4

Figure S2. Enzyme stability, and cell integrity and viability during photocatalytic conditions. S5

Figure S3. The effect on cumulative H_2 production of removing selected components from the photocatalytic system S5

Figure S4. Difference ATR-FTIR spectra of a rehydrated film of *E.coli* cells containing *Cr*HydA1, eosin Y and TEOA. S6

Table S1. Samples scheme S7

Table S2. ANOVA table for a three-level model for the oxygen-free samples after 5h S8

Table S3. ANOVA table for a three-level model for the oxygen-free samples after 24h. S8

Figure S5. H_2 photoproduction from samples representing the different combinations of variables in the 5% oxygen set S9

Table S4. ANOVA table for a three-level model for the oxygen-exposed samples after 5h. S9

Figure S6. Main effects and interactions plot for the 5h time point on the oxygen-exposed set. . S10

References S10

Experimental

General

All chemicals were purchased from Sigma-Aldrich and VWR, and used as received.
All anaerobic work was performed in an MBRAUN glovebox under argon atmosphere ($[O_2] < 1$ ppm).

Strains and plasmids

The expression vector and the gene coding for hydA1 have been kindly provided by Prof. Marc Fontecave (College de France, Paris/CEA, Grenoble). The plasmid was used to transform the BL21(DE3) *E. coli* strain that was used through the study.

Complex synthesis

The $[Fe_2(adt)(CO)_4(CN)_2](Et_4N)_2$ ($[2Fe]^{adt}$) complex was synthesized in accordance with literature protocols with minor modifications, and verified by FTIR spectroscopy.[1-4] The complex was dissolved in anaerobic potassium phosphate buffer (100 mM, pH 6.8) at 10 µg/µL concentration and used directly.

Whole-cell samples preparation.

Whole cell holo-*Cr*HydA1 samples have been prepared as previously reported. [5] Following complex insertion, the cell suspensions have been washed three times in PBS buffer (100mM NaH_2PO_4-Na_2HPO_4, 150mM NaCl, 100mM triethanolamine (TEOA)), and with a pH adjusted to 6.5 or 7.5 according to needs using H_3PO_4. The washed cell suspension was then diluted to 2 ml final volume in the same buffer to a specific OD at 600 nm, and eosin Y was added as specified in the text.

Cell integrity assay

The integrity of the cells was determined following previously reported protocols, with minor modifications.[6, 7] In short, whole-cell samples were collected after 2 h, 4 h, 8 h, 16 h and 24 h of illumination, transferred into an anaerobic glovebox and centrifuged (5000 rpm, 10 minutes) to separate a cell pellet from the supernatant. The cell pellet was subsequently resuspended in 2 ml lysis buffer (60 mM potassium phosphate buffer, pH 6.8; 1 mg / ml lysozyme, 0.1 mg / ml DNAse, and 0.2 % Triton X-100) and lysed via four freeze-thaw cycles using liquid N_2.
10 µl aliquots of the supernatant fractions and of the lysed pellets solutions were collected and transferred into glass vial with 1.8 ml of 100mM potassium phosphate buffer pH 6.8, 10 mM methyl viologen and 1% Triton X-100. The vials were sealed using rubber septa and the H_2-evolution reaction was started by rapid addition of 0.2 ml of a 1 M sodium dithionite solution (final concentration 100 mM). Glass vials were then incubated at 37 °C for 15 minutes, and hydrogen production was monitored sampling the vials' headspace and analysing it using a gas-chromatograph.

Cell viability assay

Agar plates for cell viability studies were prepared with 20 g/L LB medium and 100 mg/L ampicillin. The samples, prepared analogously to the photocatalytic samples, were either kept under light irradiation or in the dark at 30 °C. They were taken at different time points and 100 µL of the cell suspension was spread on the agar plates. The plates were incubated over night at 37 °C or over several days at room temperature.

Fluorescence microscopy

Pictures were taken on a Leica TCS SP5 confocal microscope. *E. coli* cells stained with eosin Y (see "Whole-cell samples preparation") were deposited on a microscope glass and fixed in position using agarose gel. An Argon laser at 514 nm wavelength was used for excitation, while a 535-590 nm window was used for emission detection. Pictures represent images collected on a single focus plane, as z-stacking was inapplicable due to photosensitizer bleaching significantly lowering image quality.

Hydrogen production assay.
Following eosin Y addition (see "Whole-cell samples preparation"), the cell suspension was transferred to Chromacol 6-CV glass vials and sealed with a rubber septum. The vials were transferred to a shaker at 30°C and kept in darkness for 2h before being placed under a triphosphor fluorescent white lamp. Light intensity was verified using a *Li-Cor Inc.* LI185B photometer.

Hydrogen measurements.
The headspace atmosphere was sampled at specific time-points and hydrogen content was determined using a gas-chromatograph (GC) (PerkinElmer LLC, MA, USA) equipped with a thermal conductivity detector (TCD) and a stainless-steel column packed with Molecular Sieve (60/80 mesh). The amount of H_2 produced was estimated based on the observed peak area and using a calibration curve. The latter was generated by injection of known amounts of hydrogen from vials including buffer (to reflect the photocatalytic solution) and extrapolating a linear dependency between the area of the GC peak and the amount of injected hydrogen in µmol. The operational temperatures of the injection port, the oven and the detector were 100°C, 80°C and 100°C respectively. Argon was used as carrier gas at a flow rate of 35 ml min^{-1} and nitrogen as a reference gas.

Apparent quantum yield calculations.
Apparent quantum yield was expressed as the percent ratio between the number of photons used for H_2 evolution (i.e. number of H_2 molecules produced x2) and the total amount of photons in the 400-800 nm wavelength region hitting the sample surface over the course of the experiment.
The latter value was obtained converting the light intensity, measured in lux using a *Li-Cor Inc.* LI185B photometer, into a photon flux (µmol of photons m^{-2} s^{-1}) by using the lux-to-photon flux conversion factor specific for cold fluorescent white lamps (0.014) and multiplying it by the sample surface (in m^2) and the illumination time (in s).

Data Analysis.
A multi-way analysis of variance was performed on data coming from the hydrogen production assay using the *anovan()* function in MATLAB® (Mathworks). The data vector contained the values for cumulative hydrogen production at specific time points (in µmol), whereas the grouping variables were defined as cell arrays, each containing the levels of the variable for the samples. The levels were expressed as +1 (high level), -1 (low level) and, for three-level variables, 0 (mid level).

EPR
Whole-cell samples holo-*Cr*HydA1 samples were prepared and stained as described in "Whole-cell samples preparation". After staining, several vials were collected, pelleted and re-suspended in 400 µL PBS buffer, before transferred to a quartz X-band EPR tube. Samples were either dark-incubated or illuminated anaerobically for 3h or 24h, after which they were flash frozen in liquid nitrogen. EPR spectra were recorded on a Bruker ELEXYS E500 spectrometer using an ER049X SuperX microwave bridge in a Bruker SHQ0601 cavity equipped with an Oxford Instruments continuous flow cryostat and using an ITC 503 temperature controller (Oxford Instruments). The spectrometer was controlled by the Xepr software package (Bruker). Spectra were processed using in-house scripts for the MATLAB and Qsoas[8] softwares.

FTIR
For whole-cell ATR FTIR spectroscopy, 1-2 µL of an *E. coli* suspension, prepared in PBS buffer, pH 7.5, with 100 µM eosin Y and 100 mM TEOA were deposited on the silicon crystal of an ATR cell (BioRadII from Harrick). Its hydration level was controlled using an inert gas flow carrying a buffer aerosol (10 mM Tris pH 8). Spectra were then collected with a Vertex 70v FTIR spectrometer (Bruker). All experiments were performed at ambient temperature and pressure. The light from a Schott KL2500LCD cold light source was used to induce light-dependent redox states changes.

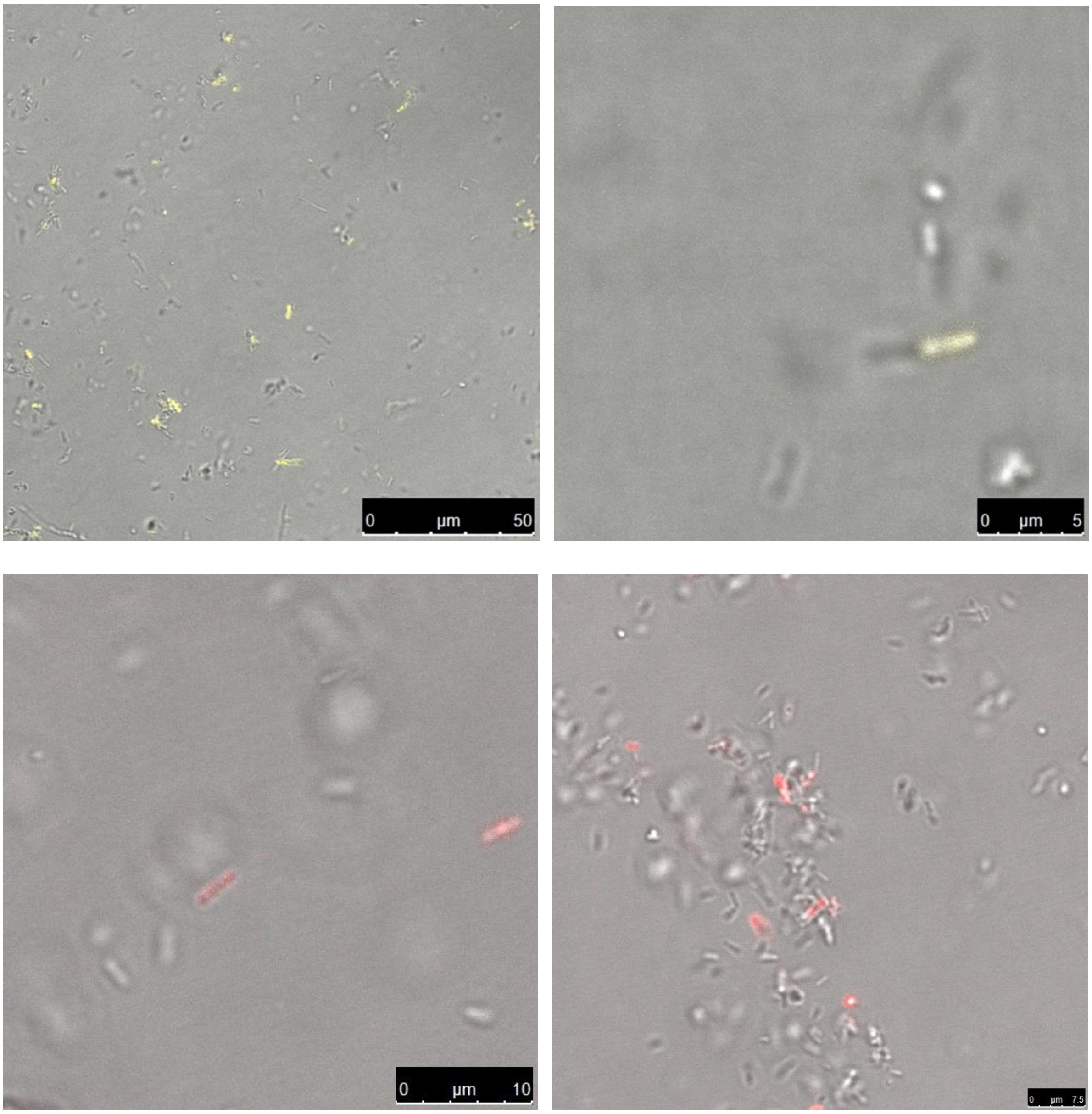

Figure S1. Fluorescence microscopy pictures of eosin Y-stained *E. coli* cells.
Pictures show single focus planes of an eosin Y-stained *E.coli* sample encased in an agar gel to hinder bacterial mobility. Fluorescence is shown in fake colours (yellow or red). Excitation wavelength = 513 nm, emission detection window = 535-590 nm.

The presence of several apparently unstained rod shapes is attributed to the presence of cells above and below the microscope focal plane and to laser-induced bleaching, which lowers image quality and prevents z-stacking of different focal planes on the same spot.

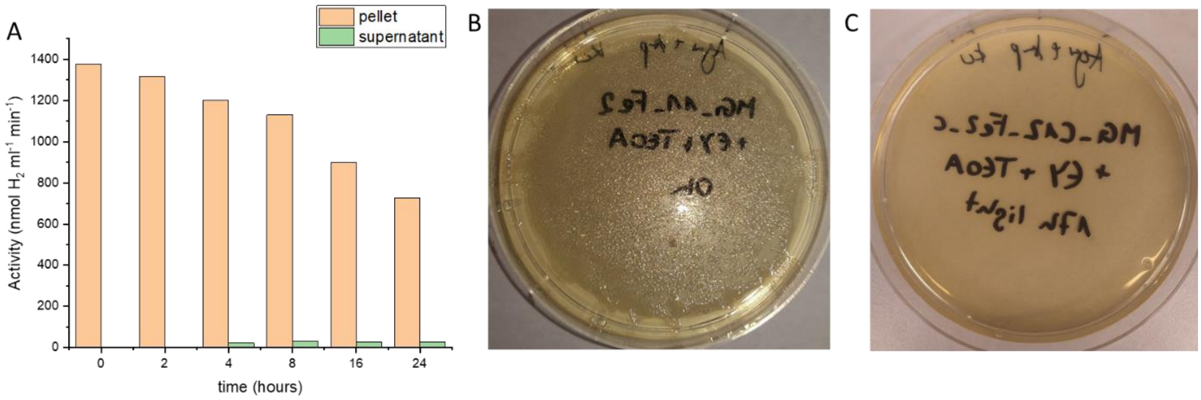

Figure S2. Enzyme stability, and cell integrity and viability during photocatalytic conditions. (**Panel A**) The [FeFe] hydrogenase activity of the whole-cell component (pellet) and supernatant at selected time-points during the photocatalytic assay. Even after 24 h of light irradiation >50% of the original activity remains, of which >95% is localized inside the cells. (**Panels B and C**) Determination of cell viability. Agar plates generated by streaking a fraction of the photocatalytic mix before (B) and after 17 h (C) of light irradiation. Multiple colonies are observed prior to irradiation, while viability appears to be diminished after 17 h.

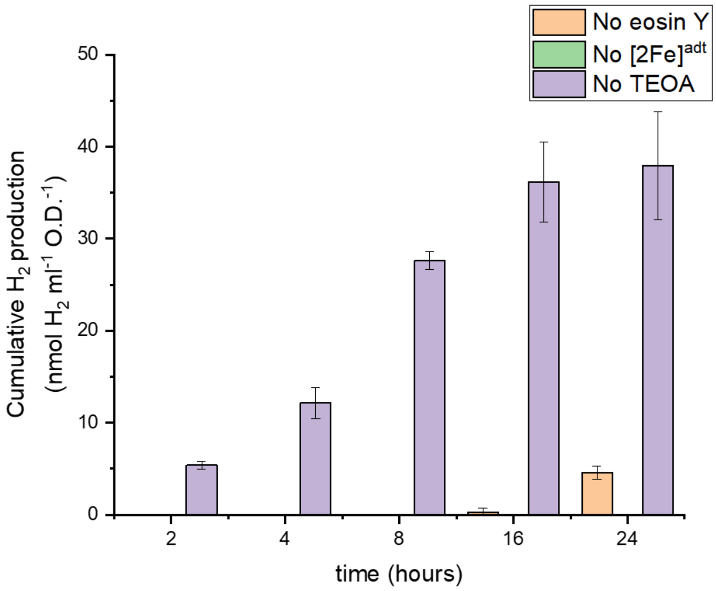

Figure S3. The effect on cumulative H_2 production of removing selected components from the photocatalytic system. H_2 production was quantified at selected time-points in samples prepared analogously to the fully functional photocatalytic system. The standard reaction mixture contained eosin Y (100 µM) and TEOA (100 mM), buffered at pH = 7.5, and cells were treated with [2Fe]^{adt} to yield the functional [FeFe] hydrogenase. However, each control lacked one of the aforementioned components (eosin Y, active enzyme or TEOA), as indicated in the figure. No H_2 production was observed for cells not treated with [2Fe]^{adt}. Trace levels of activity was observed in the absence of TEOA (purple bars), as well as eosin Y (orange bars). Note that under equivalent conditions the fully functional system with all components reaches a cumulative H_2 production of ~0.5 µmol ml^{-1} OD_{600}^{-1} of H_2 over the course of 24 h

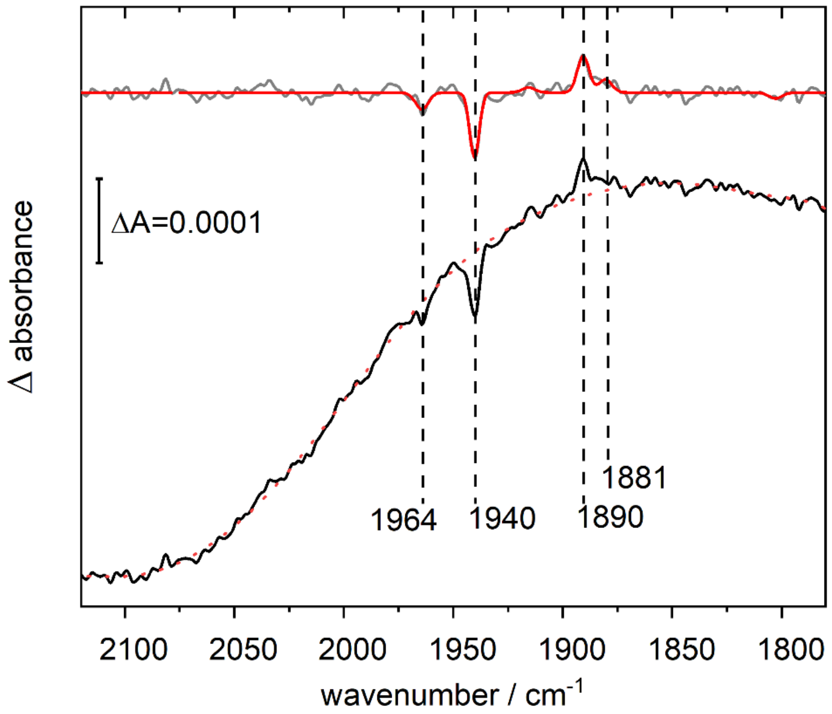

Figure S4. Difference ATR-FTIR spectra of a rehydrated film of *E.coli* cells containing *Cr*HydA1, eosin Y and TEOA. On the bottom part of the figure, a difference spectrum recorded during sample illumination (black line) is overlapped with the baseline correction applied to account for contributions coming from water (red dotted line). On the top part of the figure, the resulting baseline corrected spectrum (grey line) is overlapped with a peak fit. Wavenumber for identified bands are given as labels.

Table S1. Samples scheme. The value assigned to the variable is represented by a –1 (low value), a +1 (high value) or a 0 (middle value) and reflect the values presented in Table 1 in the main text. The same samples scheme was used for the oxygen-free and the 5% oxygen datasets.

Sample Number	**OD_{600}**	**EY**	**pH**	**Light1**	**Light2**	**LightT**
1	-1	-1	-1	-1	-1	-1
2	+1	-1	-1	-1	-1	-1
3	-1	+1	-1	-1	-1	-1
4	-1	-1	+1	-1	-1	-1
5	-1	-1	-1	+1	-1	0
6	-1	-1	-1	-1	+1	0
7	+1	+1	-1	-1	-1	-1
8	+1	-1	+1	-1	-1	-1
9	+1	-1	-1	+1	-1	0
10	+1	-1	-1	-1	+1	0
11	-1	+1	+1	-1	-1	-1
12	-1	+1	-1	+1	-1	0
13	-1	+1	-1	-1	+1	0
14	-1	-1	+1	+1	-1	0
15	-1	-1	+1	-1	+1	0
16	-1	-1	-1	+1	+1	+1
17	+1	+1	+1	-1	-1	-1
18	+1	+1	-1	+1	-1	0
19	+1	+1	-1	-1	+1	0
20	+1	-1	+1	+1	-1	0
21	+1	-1	+1	-1	+1	0
22	+1	-1	-1	+1	+1	+1
23	-1	+1	+1	+1	-1	0
24	-1	+1	+1	-1	+1	0
25	-1	+1	-1	+1	+1	+1
26	-1	-1	+1	+1	+1	+1
27	+1	+1	+1	+1	-1	0
28	+1	+1	+1	-1	+1	0
29	+1	+1	-1	+1	+1	+1
30	+1	-1	+1	+1	+1	+1
31	-1	+1	+1	+1	+1	+1
32	+1	+1	+1	+1	+1	+1

Table S2. ANOVA table for a three-level model for the oxygen-free samples after 5h. The table includes the sources of variance considered in the analysis (Source), the total amount of variation attributed to each source (Sum. Sq.), the number of degrees of freedom (d.f.), the mean squares (Mean Sq., i.e. Sum Sq. per degree of freedom), and the F-statistic and p-values (Prob>F) for each source.

Analysis of Variance

Source	Sum Sq.	d.f.	Mean Sq.	F	Prob>F
OD	270701.1	1	270701.1	21.83	0.0009
EY	287490.2	1	287490.2	23.18	0.0007
pH	68600.1	1	68600.1	5.53	0.0405
LightT	10581.4	2	5290.7	0.43	0.6641
OD*EY	109326.9	1	109326.9	8.82	0.0141
OD*pH	18022.5	1	18022.5	1.45	0.2558
OD*LightT	48488.9	2	24244.5	1.95	0.192
EY*pH	168059.5	1	168059.5	13.55	0.0042
EY*LightT	48298.2	2	24149.1	1.95	0.1931
pH*LightT	56751.1	2	28375.6	2.29	0.152
OD*EY*pH	17505.1	1	17505.1	1.41	0.2623
OD*EY*LightT	73967	2	36983.5	2.98	0.0964

Table S3. ANOVA table for a three-level model for the oxygen-free samples after 24h. The table includes the sources of variance considered in the analysis (Source), the total amount of variation attributed to each source (Sum. Sq.), the number of degrees of freedom (d.f.), the mean squares (Mean Sq., i.e. Sum Sq. per degree of freedom), and the F-statistic and p-values (Prob>F) for each source.

Analysis of Variance

Source	Sum Sq.	d.f.	Mean Sq.	F	Prob>F
OD	2955728.5	1	2955728.5	58.75	0
EY	143270.1	1	143270.1	2.85	0.1224
pH	240199.4	1	240199.4	4.77	0.0538
LightT	28143.6	2	14071.8	0.28	0.7617
OD*EY	41259.5	1	41259.5	0.82	0.3865
OD*pH	1714876.9	1	1714876.9	34.08	0.0002
OD*LightT	83302.2	2	41651.1	0.83	0.4649
EY*pH	314142.2	1	314142.2	6.24	0.0315
EY*LightT	355037.9	2	177519	3.53	0.0693
pH*LightT	324717.9	2	162358.9	3.23	0.0829
OD*EY*pH	40994.6	1	40994.6	0.81	0.3879
OD*EY*LightT	226735	2	113367.5	2.25	0.1557

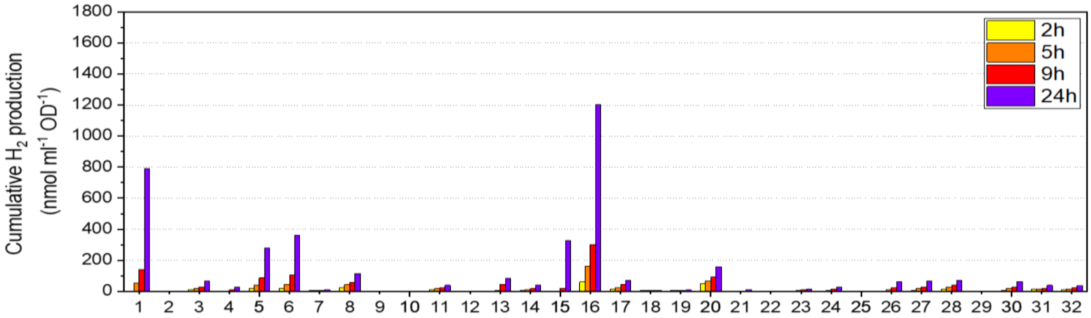

Sample Number	1	2	3	4	5	6	7	8	9	10	11	12	13	14	15	16
OD_{600}	-1	1	-1	-1	-1	-1	1	1	1	1	-1	-1	-1	-1	-1	-1
EY	-1	-1	1	-1	-1	-1	1	-1	-1	-1	1	1	1	-1	-1	-1
pH	-1	-1	-1	1	-1	-1	-1	1	-1	-1	1	-1	-1	1	1	-1
LightT	-1	-1	-1	-1	0	0	-1	-1	0	0	-1	0	0	0	0	1

Sample Number	17	18	19	20	21	22	23	24	25	26	27	28	29	30	31	32
OD_{600}	1	1	1	1	1	1	-1	-1	-1	-1	1	1	1	1	-1	1
EY	1	1	1	-1	-1	-1	1	1	1	-1	1	1	1	-1	1	1
pH	1	-1	-1	1	1	-1	1	1	-1	1	1	1	-1	1	1	1
LightT	-1	0	0	0	0	1	0	0	1	1	0	0	1	1	1	1

Sample Number

Figure S5. H_2 photoproduction from samples representing the different combinations of variables in the 5% oxygen set. Cumulative H_2 production is expressed as nmol ml^{-1} OD^{-1}. For each sample, data is shown for H_2 produced after 2 h, 5 h, 9 h and 24 h of illumination. The Sample Number (1-32) refers to a specific combination of variables as defined in the bottom table, colour coding: +1 (green); 0 (white); -1 (pink). See also Table 1 in the main text and Table S1 for additional details.

Table S4. ANOVA table for a three-level model for the oxygen-exposed samples after 5h. The table includes the sources of variance considered in the analysis (Source), the total amount of variation attributed to each source (Sum. Sq.), the number of degrees of freedom (d.f.), the mean squares (Mean Sq., i.e. Sum Sq. per degree of freedom), and the F-statistic and p-values (Prob>F) for each source.

Analysis of Variance

Source	Sum Sq.	d.f.	Mean Sq.	F	Prob>F
OD	1345.4	1	1345.37	4.66	0.0628
EY	2898.9	1	2898.87	10.05	0.0132
pH	287.8	1	287.77	1	0.3472
LightT	968.7	2	484.34	1.68	0.2461
OD*EY	1879.2	1	1879.18	6.51	0.0341
OD*pH	6635.2	1	6635.18	23	0.0014
OD*LightT	2174.6	2	1087.32	3.77	0.0703
EY*pH	2220.9	1	2220.88	7.7	0.0241
EY*LightT	1332.2	2	666.1	2.31	0.1616
pH*LightT	1247.4	2	623.72	2.16	0.1776
OD*EY*pH	4590.9	1	4590.9	15.91	0.004
OD*EY*LightT	1652.7	2	826.37	2.86	0.1153
OD*pH*LightT	648.9	2	324.45	1.12	0.3712

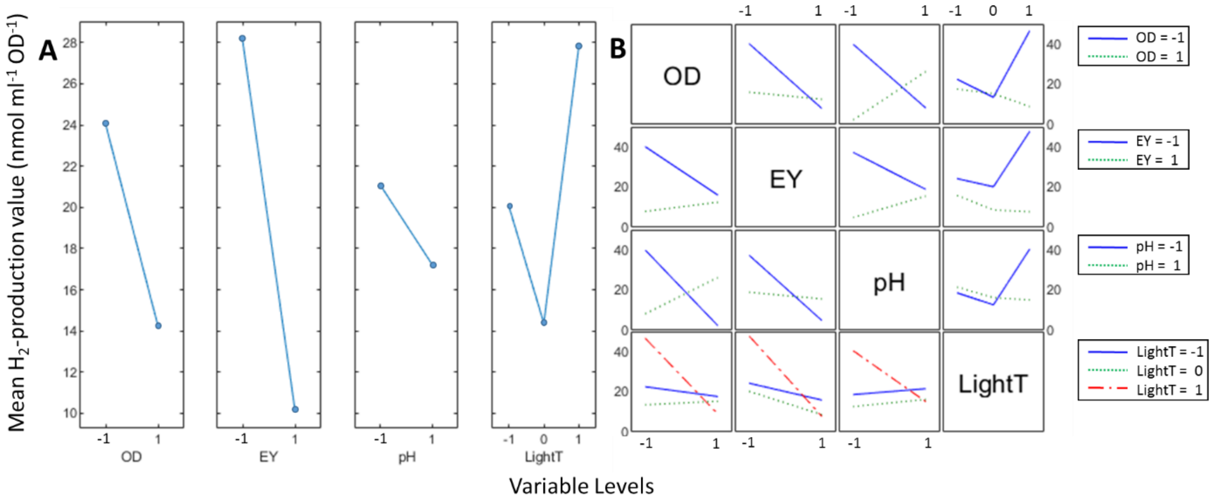

Figure S6. Main effects and interactions plot for the 5h time point on the oxygen-exposed set. The Main effects plot (panel A) visualizes the magnitude and the direction of the effect of varying the level of the single variables on the mean H_2 production. The interaction plot (panel B) shows the effect of a single variable (columns, levels indicated on the x-axes) in relation to the level of another distinct variable (rows, levels represented with colored lines as indicated in the legends).

References

1. Le Cloirec, A.; C. Davies, S.; J. Evans, D.; L. Hughes, D.; J. Pickett, C.; P. Best, S.; Borg, S., A di-iron dithiolate possessing structural elements of the carbonyl/cyanide sub-site of the H-centre of Fe-only hydrogenase. *Chem. Comm.* **1999,** *22*, 2285-2286.
2. Lyon, E. J.; Georgakaki, I. P.; Reibenspies, J. H.; Darensbourg, M. Y., Carbon Monoxide and Cyanide Ligands in a Classical Organometallic Complex Model for Fe-Only Hydrogenase. *Angew. Chem. Int. Ed.* **1999,** *38* (21), 3178-3180.
3. Schmidt, M.; Contakes, S. M.; Rauchfuss, T. B., First Generation Analogues of the Binuclear Site in the Fe-Only Hydrogenases: $Fe_2(\mu\text{-}SR)_2(CO)_4(CN)_2^{2-}$. *J. Am. Chem. Soc.* **1999,** *121* (41), 9736-9737.
4. Li, H.; Rauchfuss, T. B., Iron Carbonyl Sulfides, Formaldehyde, and Amines Condense To Give the Proposed Azadithiolate Cofactor of the Fe-Only Hydrogenases. *J. Am. Chem. Soc.* **2002,** *124* (5), 726-727.
5. Meszaros, L. S.; Nemeth, B.; Esmieu, C.; Ceccaldi, P.; Berggren, G., In vivo EPR characterization of semi-synthetic [FeFe] hydrogenases. *Angew. Chem. Int. Ed.* **2018,** *57*, 2596-2599.
6. Khanna, N.; Esmieu, C.; Meszaros, L. S.; Lindblad, P.; Berggren, G., In vivo activation of an [FeFe] hydrogenase using synthetic cofactors. *Energy Environ. Sci* **2017,** *10* (7), 1563-1567.
7. Lorenzi, M.; Ceccaldi, P.; Rodríguez-Maciá, P.; Redman, H. J.; Zamader, A.; Birrell, J. A.; Mészáros, L. S.; Berggren, G., Stability of the H-cluster under whole-cell conditions—formation of an H_{trans}-like state and its reactivity towards oxygen. *J. Biol. Inorg. Chem.* **2022,** *27* (3), 345-355.
8. Fourmond, V., QSoas: A Versatile Software for Data Analysis. *Anal. Chem.* **2016,** *88* (10), 5050-5052.
